# Assessment of Landscape Ecological Health: A Case Study of a Mining City in a Semi-Arid Steppe

**DOI:** 10.3390/ijerph16050752

**Published:** 2019-03-01

**Authors:** Zhenhua Wu, Shaogang Lei, Bao-Jie He, Zhengfu Bian, Yinghong Wang, Qingqing Lu, Shangui Peng, Linghua Duo

**Affiliations:** 1 Engineering Research Center of Ministry of Education for Mine Ecological Restoration, China University of Mining and Technology, Xuzhou 221116, China; wzhdjtc@126.com (Z.W.); lsgang@126.com (S.L.); 2 School of Environment Science and Spatial Informatics, China University of Mining and Technology, Xuzhou 221116, China; wyh3337@163.com (Y.W.); luluqingzi@163.com (Q.L.); 3 Faculty of Built Environment, University of New South Wales, Sydney 2052, Australia; baojie.he@unsw.edu.au; 4 School of Business, Shandong Normal University, Ji’nan 250014, China; pengshangui@163.com; 5 China University of Mining and Technology (Beijing), Beijing 100083, China; duolinghua@student.cumtb.edu.cn

**Keywords:** assessment of landscape ecological health, CVORE model, semi-arid Steppe, mining city

## Abstract

The ecological status of the semi-arid steppes in China is fragile. Under the long-term and high-intensity development of mining, the ecological integrity and biodiversity of steppe landscapes have been destroyed, causing soil pollution, grassland degradation, landscape function defect, and so on. Previous studies have mainly focused on ecosystem health assessment in mining areas. Landscape ecological health (LEH) pays more attention to the interactions between different ecosystems. Therefore, the ecological assessment of mining cities is more suitable on a landscape scale. Meanwhile, the existing LEH assessment index systems are not applicable in ecologically fragile areas with sparse population, underdeveloped economy, and in relatively small research areas. The purpose of this study was to construct a LEH assessment index system and evaluate the LEH of a mining city located in a semi-arid steppe. Xilinhot is a typical semi-arid steppe mining city in China. The contradictions between the human, land and ecological environment are serious. A new model Condition, Vigor, Organization, Resilience, and Ecosystem (CVORE) model was constructed that integrated five subsystems (services) from the perspectives of ecology, landscape ecology, mining science, and geography. This study used the CVORE model to systematically evaluate the LEH in Xilinhot city in terms of five LEH levels, including very healthy, healthy, sub-healthy, unhealthy and morbid landscape. Research results show that the areas of the very healthy, healthy, sub-healthy, unhealthy and morbid landscapes are 13.23, 736.35, 184.5, 66.76 and 20.63 km^2^, respectively. The healthy landscapes area accounts for 72.08% and most grasslands are healthy. The sub-healthy landscapes are mainly located around areas with higher disturbances due to human activities. The morbid or unhealthy landscapes are concentrated in the mining areas. The proposed CVORE model can enrich the foundations for the quantitative assessment of Landscape Ecological Health of Mining Cities in Semi-arid Steppe (LEHMCSS). This study provided a new LEH assessment approach (CVORE model), which can support landscape ecological restoration, ecological environmental protection and urban planning of the semi-arid steppe mining cities.

## 1. Introduction

Grasslands represent the largest terrestrial ecosystem and an important component of the global natural ecosystem. They play an important role in animal husbandry development, wind and sand control, soil and water conservation, biodiversity protection and ecological balance [1,2]. Natural ecosystems provide both the material basis and the ecological services for the subsistence and the development of human societies [3,4]. A mining city is generally developed from its rich mineral resources. Depending on its local resources, the main development of a mining city is usually based on raw materials or raw energy outputs. Mining cities are an important mineral supply base for the process of modernization, which make an important contribution to the support and promotion of the economic development of a country [5]. However, the upsurge of coal industrialization and urbanization, together with the expanded breadth and intensity of human activities, have changed semi-arid steppe ecosystems at an unprecedented speed and scale, leading to regional ecosystem degradation and significant threats to the survival and development of human society. Meanwhile, mining areas have typically become the most deteriorated ecosystems in the world’s terrestrial biosphere [6]. The long-term high-intensity development of open-pit coal mines has not only brought tremendous changes to the structure of surface landscapes, but also affected the material circulation and energy flow of landscapes, resulting in significant changes in regional climate, soil, biodiversity, hydrology and water resources, and profoundly impacting regional ecological processes [7,8,9,10]. In order to tackle the impacts of mining areas landscape ecosystem degradation on regional sustainable development in semi-arid steppe area, increasing attention has been paid to how to monitor, assess, and regulate the state and sources of risk, as well as the safety or sustainability degree of landscape ecological health (LEH).

LEH originates from the concepts of natural health [11,12], land health [13], ecological medicine [14] and ecosystem health [15]. Ferguson [16] extended the concept of health to the landscape scale for the first time, considering landscape health as a “dynamic balance” state, where the regulation and feedback mechanism maintains the automatic regulation function of the whole landscape. LEH refers to the stability and sustainability of the rich ecosystem services provided by different types of ecosystems within a certain space-time range and on the premise of maintaining their own health [17]. The concept of LEH has been proposed to explore the health problems of landscape that are seriously polluted and degraded, or may even disappear, under the disturbance of human activities [18]. LEH assessment will provide scientific guidance and suggestions for regional ecological environment protection and planning.

Current research focuses on ecosystem health assessment in mining areas [19,20,21,22]. However, we need to pay more attention to the health of the surrounding ecosystems under the influence of mining ecosystems. The scale of landscape ecology is higher than that of ecosystem ecology. LEH pays more attention to the interaction between different ecosystems. The semi-arid steppe mining city is composed of grassland, mining area, city, and other ecosystems. Therefore, the ecological assessment of the semi-arid steppe mining cities is more suitable at the landscape scale. LEH assessments generally include two methods: The indicator species method, and the index system method. The indicator species method mainly describes the LEH status based on the number, biomass and some physiological and ecological indicators of key species, indicator species and environmentally sensitive species in an ecosystem. The key species are the species whose disappearance or weakening can cause fundamental changes in the whole community and ecosystem. Indicator species are those that can represent the characteristics of a certain stage in the succession series of communities or can be used to judge the types and characteristics of the natural environment. Environmentally sensitive species can reflect whether certain substances in the environment exceed the standard, such as soil salt content, heavy metal content in the soil, etc. The index system method establishes an index system for quantitative assessment of LEH based on the characteristics of landscape ecology and its service functions, such as landscape fragmentation, landscape connectivity decline, drought, salinization, desertification, low vegetation coverage, poor ecological resilience after disturbance, high heterogeneity of ecosystem services, and so on in the semi-arid steppe mining city [17]. The indicator species method is mainly used for natural ecosystems, including forests, extreme environments, and wetlands, and is based on evaluations of indicator species, key species, and endemic species. This method does not consider human activity factors, so it is not suitable for LEH in regions experiencing intensive human activity such as mining cities [23]. Because the landscape is a regional mosaic of multiple ecosystems, it is difficult to find appropriate indicator species (groups) to monitor landscape health status. Therefore, the index system method is commonly used to assess LEH [17]. However, the majority of existing index systems select economic, social, industrial, environmental and other statistical data based on administrative divisions, which are not applicable in ecologically fragile areas with sparse population, underdeveloped economy, and in relatively small research areas.

The goals of this article are thus to: (1) construct LEH assessment index system for a semi-arid steppe mining city; (2) Assess the LEH status of a semi-arid steppe mining city; (3) highlight the properties of a mining city in semi-arid steppe to provide other similar cities with the experience of LEH assessment.

## 2. Materials and Methods

### 2.1. Assessment Index System for Mining City Landscape Ecological Health in Semi-Arid Steppe

Adopting a system sustainability perspective, Costanza and Rapport put forward the Index of Ecological Health (EH), composed of three dimensions: ecosystem vigor (V), organization (O), and resilience (R), following the formula EH = V × O × R [24]. The American Grassland Society put forward that grassland health should be taken as a criterion to evaluate grassland base conditions [25]. The contents of nutrients, salts, and heavy metals in soil directly affect many factors such as surface plant communities. Soil condition is a clear sign of grassland base condition change and an important index for grassland LEH assessment [26]. Ecosystem services have always been important in providing an indication of ecological health in human-nature coupled systems [27]. Costanza [28] proposed a healthy ecosystem as a primary design goal for ecological engineering, which sustainably provides a series of valuable ecosystem services. So, it is necessary to figure out the relation between ecological health and the provision of ecosystem services and to determine the ecosystem malfunctions related to these services in an ecological health assessment [29]. At the same time, landscapes generate a wide range of valuable ecosystem services. It is thus considered important to focus on how changes in ecosystem services interact with various land use types, in addition to the effect of human activities, in order to gain a more comprehensive understanding of LEH [30,31].

**Figure 1:**
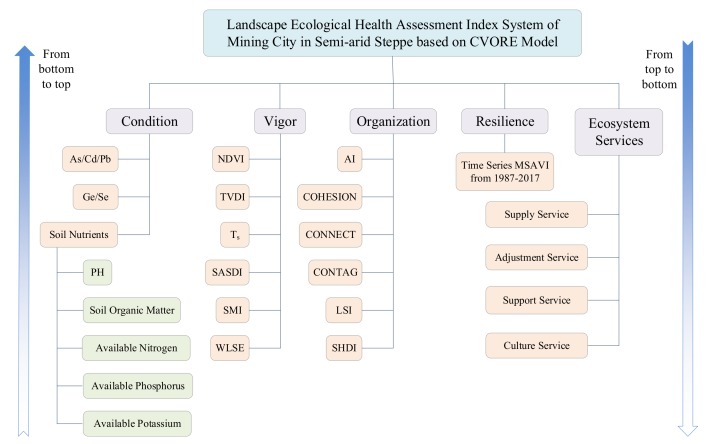
Landscape ecological health assessment index system.

Therefore, it is better to consider the Condition (C), Vigor (V), Organization (O), Resilience (R), and Ecosystem services (E) together to evaluate the LEH in semi-arid steppe areas. The comprehensive assessment system integrating these factors (i.e., CVORE model) was proposed and constructed in this paper to systematically evaluate the LEH of Xilinhot, a typical mining city. Combined with the characteristics of the mining city in semi-arid steppe, the related indexes were selected, as illustrated in Figure 1. The formula of the Landscape Ecological Health of Mining City in Semi-arid Steppe (*LEHMCSS*) is the following:

(1) $$LEHMCSS=\sqrt[{5}]{C\times V\times O\times R\times E}$$

where *LEHMCSS* is the physical health of the landscape ecology of spatial entities for mining city in a semi-arid steppe; and *C*, *V*, *O*, *R* and *E* refer to the condition, vigor, organization, and resilience, ecosystem services of spatial entities, respectively. All physical quantities in this study are normalized into dimensionless spatial entities.

#### 2.1.1. Indexes Selection for Condition

The Condition can be expressed as a combination of factors such as atmosphere, land, and loci of the ecosystem. The Condition is an evaluation of the land boundary pasture interface process, reflecting the matching degree between them. Range sites are the natural environmental basis for the existence of ecosystems, providing climatic conditions and nutrient requirements for vegetation growth. The range sites-forage coupling pattern determines the grassland health level [32], which depends on the occupation pattern of pasture, and, to a certain extent, can be understood as the niche of pasture. Soil Nutrient (*SN*) is a key index to measure the restoration and maintenance of the ecological functions of degraded ecosystems. Common chemical elements in the mining area, which are harmful to the human body, may include lead, arsenic, and cadmium [33], while both germanium and selenium have beneficial effects on human health, such as immunity regulation and resistance enhancement. In view of this, the following indexes were used for the evaluation of the grassland Condition: *SN*; *Cd*/*Pb* content (as soil heavy metals); *As* content (as a soil harmful substance); and *Ge*/*Se* content (as soil beneficial substances). The formulas of the Condition used are the following (2)–(5):

*C* = *w_sn_* × *SN* + *w_shm_* × (*w_cd_* × *Cd* + *w_pb_* × *Pb*) + *w_shs_* × *As* + *w_sbs_* × (*w_ge_* × *Ge* + *w_se_* × *Se*) (2)

*w_sn_* + *w_shm_* + *w_shs_* + *w_sbs_* = 1 (3)

*w_cd_* + *w_pb_* = 1 (4)

*w_ge_* + *w_se_* = 1 (5)

where *C* is the value of the basic Condition; *SN* is the value of Soil Nutrients; and *Cd*, *Pb*, *As*, *Ge* and *Se* are the soil content values of cadmium, lead, arsenic, germanium, and selenium, respectively. *w_sn_*, *w_shm_*, *w_cd_*, *w_pb_*, *w_shs_*, *w_sbs_*, *w_ge_* and *w_se_* are the weights of *SN*, heavy metal content in soil, *Cd*, *Pb*, Content of soil harmful substances, Content of soil beneficial substances, *Ge* and *Se*, respectively.

#### 2.1.2. Indexes Selection for Vigor

Vigor refers to the energy or activity of an ecosystem. Vigor is measured by the total amount or efficiency of material production and energy fixation in ecosystems. Photosynthetic rate, photosynthetic products, and aboveground biomass were selected for evaluation. Natural ecosystem Vigor is generally characterized through vegetation productivity indicators. As vegetation production is significantly positively correlated to the Normalized Difference Vegetation Index (NDVI) [34], the NDVI is usually chosen as an indicator to measure the vigor of natural ecosystems. High wetness, lower temperatures, and low conductivity indicate better functioning landscape segments and areas that are stabilized. On the contrary, areas displaying low wetness, high temperatures, and high water conductivity belong to landscape segments that are disturbed and represent localities at risk [35]. Meanwhile, heat island poses significant challenges to ecosystems, i.e., energy and water use, human thermal comfort, health and ecosystem balance [36]. Moreover, changes of landscape types are responsible for the spatial distribution of land surface temperature [37]. The greater the humidity, the lower the Temperature Vegetation Drought Index (TVDI) [38] value. The higher the temperature, the higher the surface Temperature (*T_s_*) [39] value. The higher the conductivity, the higher the Salinization Monitoring Index (SMI) [40] value. Therefore, TVDI, *T_s_*, and SMI were selected as indicators for measuring the Vigor of natural ecosystems. Desertification studies show that, with the aggravation of desertification, the surface vegetation is seriously damaged and biomass and surface vegetation coverage are reduced, resulting in a decrease in the Vigor of the landscape ecosystem. Therefore, Semi-Arid Steppe Desertification Index (SASDI) [41] was chosen as an indicator to measure the Vigor of natural ecosystems. Water Loss and Soil Erosion (WLSE) is an important surface process. Landscape elements act on the entire process of WLSE, which includes an in-situ detachment of soil materials, as well as their transport and deposition along the surface. The spatial allocation of the landscape elements has a significant impact on WLSE. Runoff erosion effects are different under different vegetation patterns. The spatial organization of bare areas and vegetation on the slope surface has a significant impact on the prediction of runoff and erosion [42]. Open-pits and dumps have seriously changed aspects like the original topography, vegetation cover, and the water system. Therefore, WLSE was selected as an indicator to measure the Vigor of natural ecosystems. According to the Digital Elevation Model (DEM), soil type, land use, and meteorological data, and topographic parameters of the watershed were extracted from DEM using the TOPAZ software. The GeoWEPP model, based on physical processes and coupling WEPP and GIS, was used to simulate the temporal and spatial distribution characteristics of WLSE in the study area. In synthesis, *NDVI*, *TVDI*, *SASDI*, *SMI*, *T_s_*, and *WLSE* were used to evaluate grassland Vigor. The formulas used for Vigor are the following (6) and (7):

*V* = *w_ndvi_* × *NDVI* + *w_ts_* × *T_s_* + *w_tvdi_* × *TVDI* + *w_smi_* × *SMI* + *w_sasdi_* × *SASDI* + *w_wlse_* × *WLSE* (6)

*w_ndvi_* + *w_ts_* + *w_tvdi_* + *w_smi_* + *w_sasdi_* + *w_wlse_* = 1 (7)

In the formulas, *V* represents Vigor; *NDVI* is the value of Normalized Difference Vegetation Index; *TVDI* is the value of Temperature Vegetation Drought Index; *SASDI* is the value of Semi-Arid Steppe Desertification Index; *SMI* is the value of Salinization Monitoring Index; *T_s_*, is the value of surface Temperature; and *WLSE* is value of Water Loss and Soil Erosion. *w_ndvi_*, *w_ts_*, *w_tvdi_*, *w_smi_*, *w_sasdi_*, *w_wlse_* represent the weights of *NDVI*, *T_s_*, *TVDI*, *SMI*, *SASDI*, and *WLSE*, respectively.

#### 2.1.3. Indexes Selection for Organization

Organization refers to the composition and structure of ecosystem species and the relationship between them. It reflects the optimization ability of ecosystem structure and function and reflects the stability of the ecosystem structure. It is acknowledged that spatial patterns are essential influencing factors in the management of ecosystem processes at a landscape scale [43]. In this way, ecosystem Organization is determined by landscape patterns in relation to landscape spatial heterogeneity and connectivity [30]. The long-term, high-intensity development of mining industry resulted in fragmentation and connectivity loss of semi-arid steppes. For these reasons, the following indices were used to evaluate grassland organizational strength: *AI* (Aggregation index); *COHESION* (Patch cohesion index); *CONNECT* (Connection index); *CONTAG* (Contagion index); *LSI* (Landscape shape index); and *SHDI* (Shannon’s diversity index). These landscape pattern indices were selected for their landscape ecological significance [44] (Table 1). The Organizational strength was calculated using the following equations (8) and (9):

*O* = *w_ai_* × *AI* + *w_cohesion_* × *COHESION* + *w_connect_* × *CONNECT* + *w_contag_* × *CONTAG* + *w_lsi_* × *LSI* + *w_shdi_* × *SHDI* (8)

*w_ai_* + *w_cohesion_* + *w_connect_* + *w_contag_* + *w_lsi_* + *w_shdi_* = 1 (9)

In the formulas, *O* is the value of Organization; *AI* is the value of Aggregation Index; *COHESION* is the value of Patch Cohesion Index; *CONNECT* is the value of Connection Index; *CONTAG* is the value of Contagion Index; *LSI* is the value of Landscape Shape Index; and *SHDI* represents the value of Shannon’s Diversity Index. *w_ai_*, *w_cohesion_*, *w_connect_*, *w_contag_*, *w_lsi_*, and *w_shdi_* represent the weights of *AI*, *COHESION*, *CONNECT*, *CONTAG*, *LSI*, and *SHDI*, respectively.

#### 2.1.4. Indexes Selection for Resilience

Resilience is the ability of the system to gradually recover when an external pressure disappears. A healthy landscape ecosystem that is resistant and resilient to external disturbances can keep species composition and productivity relatively stable in the long period [45]. In this study, Trend Line Analysis was used to simulate the spatial trend of Modified Soil Adjusted Vegetation Index (MSAVI) [46] in the study area. This method estimates the ascending or descending trend of vegetation cover in time series, the spatial distribution pattern change, and the turning or sudden change of vegetation cover in some time series, by using the least square method. This method can effectively simulate the change trend of each phase element, reflecting the spatial change characteristics of vegetation growth in different periods, so as to indicate the spatial distribution of the vegetation restoration ability after disturbance. The formula used for Resilience is the following (10):

(10) $$R=\theta_{slope}=\frac{n\times \sum_{j=1}^{n}j\times MSAVI_{j}-\sum_{j=1}^{n}j\sum_{j=1}^{n}MSAVI_{j}}{n\times \sum_{j=1}^{n}j^{2}-{\left(\sum_{j=1}^{n}j\right)}^{2}}$$

In the formula, *R* is the value of Resilience; *θ_slope_* is the slope of the trend line; *n* is the cumulative year for monitoring (with *n* = 18); *MSAVI* is the Modified Soil Adjusted Vegetation Index, and *MSAVI_j_* is the average *MSAVI* in *j*.

#### 2.1.5. Indexes Selection for Ecosystem Services

The ecosystem services value model and the ecological remote sensing information model were used to construct the ecosystem services of the study area (Equation (10)). The landscape types, the NDVI index and the Fractional Vegetation Cover (VFC) [47] in the study area were measured by remote sensing, establishing a quantitative remote sensing model for ecological assets assessment. The ecosystem services coefficients for each land use type were determined to refer to the ratio among the average ecosystem services’ values of different ecosystem types calculated by Costanza et al. [48], Xie et al. [49], and Liu [50]. According to the status of ecosystem services in the study area, the ecosystem services of the study area were assigned the following coefficients in the range 0-1, taking the Grassland Landscape as a standard: Open-Pit Landscape (0); Internal Dumping Landscape (0.47); External Dumping Landscape (0.55); Mining Construction Land Landscape (0.12); Town Construction Land Landscape (0.36); Industrial and Storage Land Landscape (0.24); Greenbelt Landscape (0.82); Under Construction Landscape in Town (0.43); Grassland Landscape (0.59); Farmland Landscape (0.56); Degraded Landscape of Agricultural and Livestock Husbandry (0.46); Water Landscape (1.0); Railway Landscape (0.51); Road Network Landscape (0.51); and High Vegetation Cover Area Landscape (0.95). The Ecosystem services (E) in the study area can be expressed as:

(11) $$E=\sum_{i=1}^{n}\sum_{j=1}^{n}\frac{\left(\frac{NDVI_{j}}{NDVI_{mean}}+\frac{VFC_{j}}{VFC_{mean}}\right)}{2}\times V_{cj}\times S_{ij}$$

In this formula, *E* is the ecosystem services; *i* represents the first *i E* of the *c* ecosystem; *V_cj_* represents the unit area value of the *i E* type of the *c* ecosystem; *j* represents the number of pixels distributed in the category *c* ecosystem in a certain area; *S_ij_* is a given constant area projection representing the area of each pixel; *NDVI* is the Normalized Difference Vegetation Index; *VFC* is the Fractional Vegetation Cover; *NDVI_mean_* and *VFC_mean_* are the mean values of NDVI and of the vegetation coverage of category *c* ecosystems in the region, respectively; *NDVI_j_* and *VFC_j_* are the NDVI and vegetation coverage of *j* pixels, respectively.

### 2.2. Study Area

The Xilinguole steppe is the core part of the temperate steppe in Eurasia. It is located in the transition zone from forest to desert grassland and is the core area of the northern sand control zone. The Xilinguole steppe is famous for its complete prairie type. It is rich in grassland biodiversity and provides abundant genetic resources for human beings. It has important research value in China and even in the world [51]. The Xilinguole steppe is one of the four natural grasslands in China and the only national grassland nature reserve in China that has been included in the United Nations Human and Biosphere Protection Network [52]. Located in the hinterland of Xilinguole steppe, Xilinhot is a typical mining city with simultaneous development of coal, petroleum, heavy metals and other mineral resources. Under the long-term multiple driving forces of human disturbance and natural disasters, such as high-intensity energy development, urban expansion, industrial development and overgrazing, the landscape pattern, process and function of grassland have gradually changed, and the health status of landscape ecology is declining. The contradiction between the human, land and ecological environment is serious. There are 248 mining cities in China [53]. Xilinhot is a typical semi-arid steppe mining city developed by the open-pit mining method. Therefore, taking Xilinhot as an example, an in-depth study of the LEH of mining city has important theoretical and practical significance for region sustainable and healthy development.

**Figure 2:**
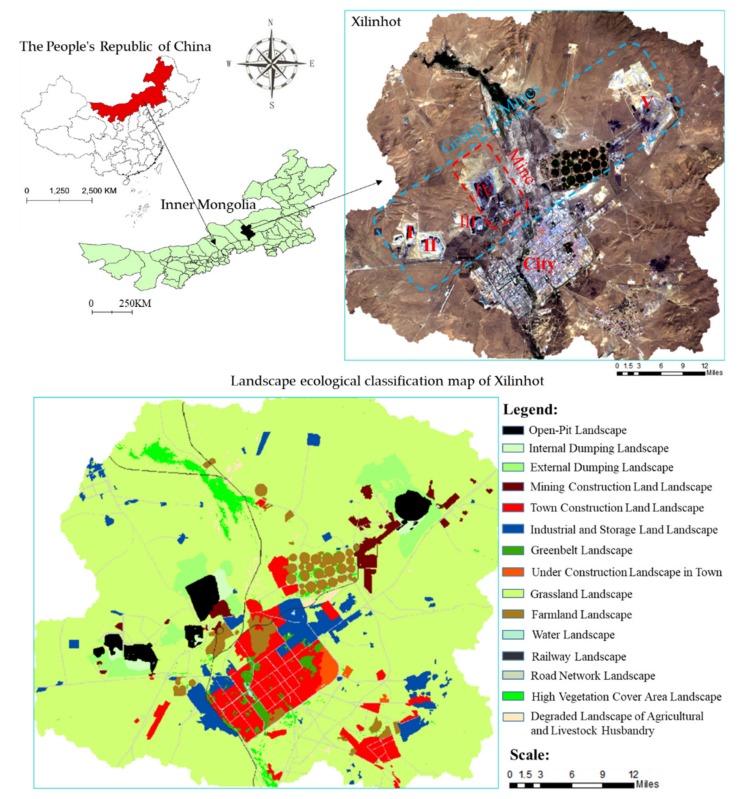
Location of the Research Area. I: Open-pit Germanium Mine; II: West No. 2 Open-pit Mine; III: West No. 3 Open-pit Mine; IV: No. 1 Open-pit Mine; V: East No. 2 Open-pit Mine.

Xilinhot is located in the Xilinguole League of the Inner Mongolia Autonomous Region of China. The location of the study area and its landscape ecological classification results are shown in Figure 2. The mining city of Xilinhot belongs to the Mengdong coal and electricity power base, which is one of the 16 large-scale coal and electricity power bases in China. The geographical coordinates range are: latitude 43°02′~44°52′ N; longitude 115°18′~117°06′ E, with an elevation of 970~1202 m. The area is located in the mid-latitude westerlies zone and belongs to the semi-arid continental climate in the middle temperate zone. The average annual rainfall is 294 mm, with an average annual evaporation capacity of 1794 mm. Evaporation far exceeds precipitation, which leads to severe desertification and salinization in the area, as shown in Figure 2. Strong winds occur in spring, with a predominant southwestern wind direction and a wind speed of 2.1–8.4 m/s, with an average speed of 3.5 m/s; the instantaneous maximum wind speed is 36.6 m/s. The maximum permafrost depth is 2.89 m, with a frost-free period of 122 days. The Xilin River is the largest river in the coalfield, with a total length of 268 km; it is currently a seasonal river. The coalfield a is oriented along the direction northeast-southwest; it has a length of 45 km, an average width of 7.6 km from south to north, a planned area of about 423 km^2^, and holds about 22.4 billion tons of total coal resources.

### 2.3. Data Source and Processing

From October to May the area is normally covered with snow. Moreover, the vegetation has not yet grown in June, while it gradually declines in September. For these reasons, the research was performed in the summer period. As cloud coverage has in some cases made Landsat data unavailable, eighteen Landsat data sets (1987/09/01, 1989/08/05, 1991/08/11, 1992/08/29, 1995/07/21, 1997/08/27, 2000/07/10, 2002/07/08, 2004/08/14, 2005/08/17, 2007/07/06, 2008/07/08, 2009/08/12, 2010/08/31, 2011/08/02, 2014/07/25, 2015/07/28, and 2017/07/17) were selected and downloaded from the US Geological Survey website (https://glovis.usgs. gov/). Landsat 5 data were used for the period from 1987 to 2011, while Landsat 8 data were used for the period after 2014. The Landsat stripe number is 124/029. Radiation calibration, FLAASH atmospheric correction, image registration, and image clipping were preprocessed using the remote sensing image processing software ENVI 5.1. Supervised classification and visual correction methods were used to obtain land use maps with the support of ENVI 5.1 and ArcGIS 10.3. The research area was divided into 15 categories (see Figure 2 for details). The spatial distribution and gradient change map of landscape pattern indices were made by using the Fragstats moving window method. In addition, according to the band ratio derived from band 1 to band 10, the NDVI, T_s_, VFC, TVDI, surface Albedo [54], MSAVI, SASDI; Salinity Index (SI) [55]; SMI were calculated. The calculation of *SASDI* is shown in Formula (12). Soil type data in the study area were purchased from the National Geographic monitoring platform (http://www.dsac.cn/). Meteorological data were downloaded from the China Meteorological Data Network (http://data.cma.cn/):

*SASDI* = *Albedo* − *a* × *MSAVI* (12)

In the formula, *a* is the vegetation line of the Albedo-MSAVI feature space.

### 2.4. Sampling, Testing and Spatial Modeling of Soil Samples

**Figure 3:**
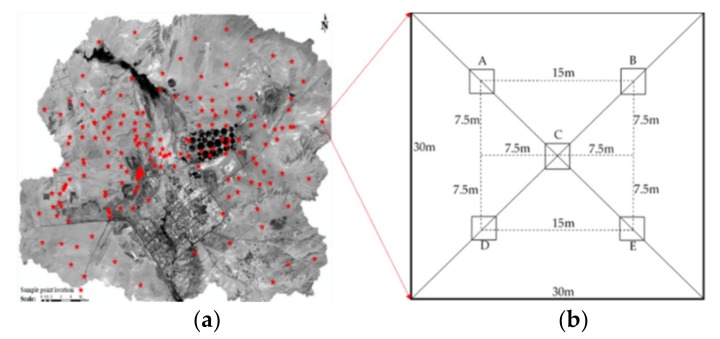
Spatial distribution of sample plots and Layout of the quadrat. (**a**) Spatial distribution of sample plots; (**b**) Layout of the quadrat.

Soil samples were collected from July to August 2017. The sample distribution is shown in Figure 3a; the total number of sample points was 174. The Galaxy 1 RTK measurement system was used for positioning. The sampling depth was in the range of 0~20 cm. Each sample was composed of four subsamples from the center point and the surrounding area (see Figure 3b). Trend surface analysis, kriging interpolation, and cross-validation were carried out on the ArcGIS 10.3 platform. Map editing was performed to create spatial distribution maps of SN, soil heavy metals, and soil As/Se. SN was constructed by soil pH, organic matter, and available nitrogen, phosphorus, and potassium.

### 2.5. Determination of Indicator Weights

Index weight is very important for LEH assessment. The entropy weight method was used to determine the relative weights of each index. The entropy weight method is used to determine the indicator’s weight of assessment of LEH in mining city based on the amount of available information provided by each indicator [56]. Its principle is: the greater the value variation of an index, the smaller the information entropy, the greater the amount of information provided by the index, the greater the corresponding weight, otherwise, the smaller the weight. According to the variation degree of each index, the weight of each index can be calculated objectively, which provides a basis for the comprehensive evaluation of multiple indexes. According to the definition of entropy, the steps of calculating the entropy of *n* indexes of *m* evaluation objects are:

(1) Standardizing the initial indicators

The indicators that included both positive and negative aspects are required to be standardized using the normalization range method so that the values of all indicators ranged from 0 to 1. The computational formulas are as follows:

Positive Indicator: *PI* = (*pi* − *pi_min_*)/(*pi_max_* − *pi_min_*) (13)

Negative Indicator: *NI* = (*ni_max_* − *ni*)/(*ni_max_* − *ni_min_*) (14)

where *PI*&*NI* is the standardized value of each index; *pi&ni* is the initial value of each index; *pi_max_*&*ni_max_* and *pi_min_&ni_min_* are the maximum value and minimum value of each index.

(2) Calculating the entropy value of each index. The formula is:

(15) $$E_{i}=-\sum_{X=1}^{m}B_{iX}\ln B_{iX}/\ln m$$

where $B_{iX}=v_{iX}/\sum_{X=1}^{m}v_{iX},$ *v_iX_* is *X*(*X* = 1, 2, …, *m*) object *i* (*i* = 1, 2, …, *n*) the magnitude of the index.

The entropy weight *W* and weight *w_i_* of evaluation index are calculated by:

*W* = (*w_i_*)_1×*n*_ (16)

where $w_{i}=\left(1-E_{i}\right)/\left(n-\sum_{i=1}^{n}E_{i}\right),$ and satisfy $\sum_{i=1}^{n}w_{i}=1.$

## 3. Results

### 3.1. Condition

**Figure 4:**
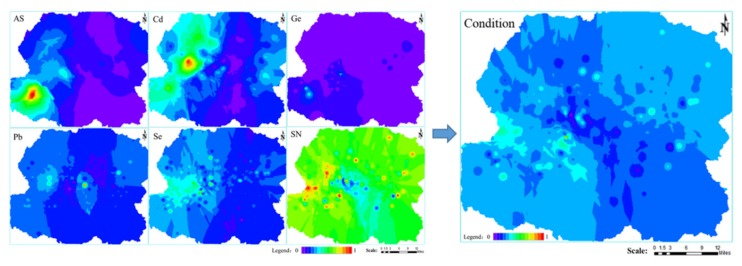
Spatial distribution map of Condition.

As shown in Figure 4, the higher the value in the figures, the higher the content of each index. The areas with the most serious As pollution are distributed around the germanium mine, the West No. 2 open-pit mine and its surrounding areas, and the No. 1 Open-pit Mine and its surrounding areas. The most serious Cd pollution areas are distributed in the pasture and in the dumps along the northwest direction of the No. 1 Open-pit Mine, and in the germanium mine and its surrounding area. The area that is most polluted by Pb lays northwest of the No. 1 Open-pit Mine and southwest of the farm. Se is mainly distributed northwest of the No. 1 open-pit mine, the West No. 2 Open-pit Mine, and the West No. 3 Open-pit Mine. The most widely distributed area of Ge is clearly located in the Open-pit Germanium Mine and its surrounding area. The highest concentrations of SN are located to the pasture north of the No. 1 Open-pit Mine, while the areas with lower nutrient concentrations are distributed between the No. 1 Open-pit Mine and the farmland. In general, the areas with better soil quality are located in the farmland south of the No. 1 Open-pit Mine and in the pasture north of the Open-pit Germanium Mine. The soil quality of the mining area and its surrounding area is poor.

### 3.2. Vigor

**Figure 5:**
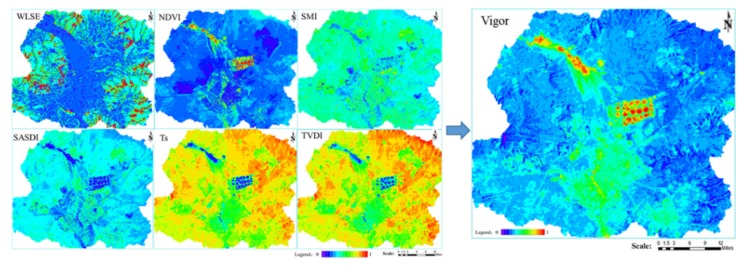
Spatial distribution of Vigor.

As shown in Figure 5, the higher the value in the figures, the higher the value of each index. The areas with higher NDVI values are mainly wetlands, cultivated land, and urban green space in the Xilin River Basin, while the areas with lower NDVI values are mainly open-pits and urban construction land. Desertification and salinization are mainly distributed in the areas around the salt marsh wetlands and in areas where human activities are frequent. The areas with higher temperature and drought are mainly around the East No. 2 Open-pit Mine, while the areas with lower temperature and higher humidity are mainly located in the wetland in the Xilin River Basin and in cultivated land. The degree of WLSE reflects the topography and the soil stability of the study area. The edge of the open-pit and the slope of the dump are the areas with the greatest risk of WLSE. In general, the areas with higher Vigor are mainly the wetlands in the Xilin River Basin and the cultivated land, while the areas with lower Vigor are mainly the open-pits and their surrounding areas. There is a strong correlation between landscape types and Vigor.

### 3.3. Organization

**Figure 6:**
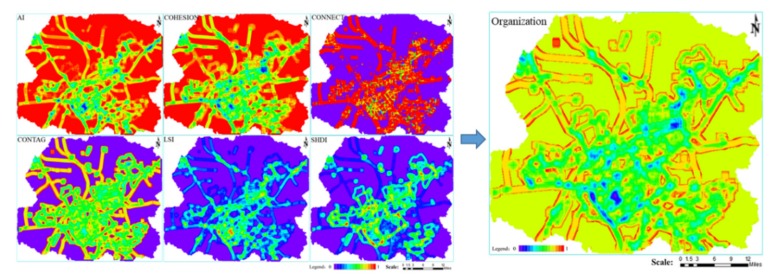
Spatial distribution map of the Organization.

As shown in Figure 6, the higher the value in the figures, the higher the value of each index. From the six indexes of AI, COHESION, CONNECT, CONTAG, LSI, and SHDI, the open-pit mines and towns lead to landscape fragmentation. On the one hand, the road divides the grassland landscape and accelerates the fragmentation of the regional landscape. On the other hand, the road acts as a corridor and enhances the connectivity for human activities.

### 3.4. Resilience

**Figure 7:**
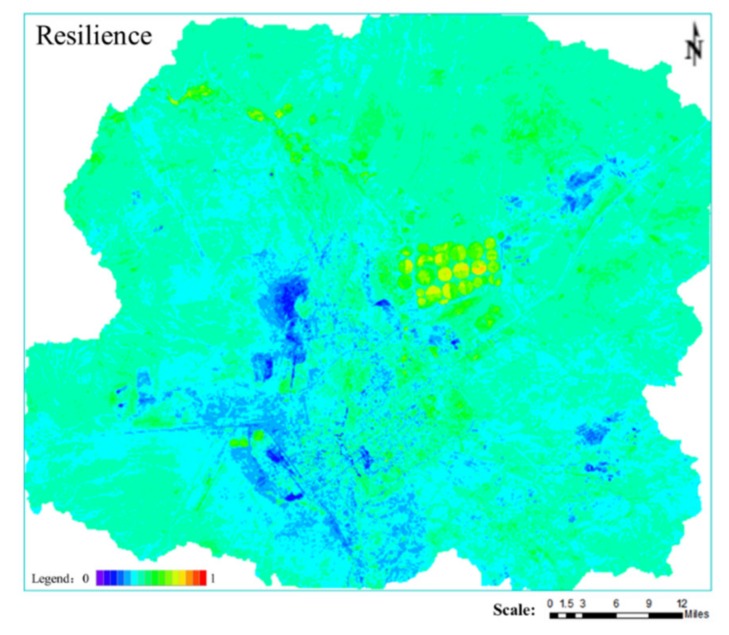
Spatial distribution map of Resilience.

In Figure 7, the higher the value, the greater the resilience. Figure 7 shows that the areas with lower resilience values are mainly located in the open-pit and in the industrial storage land. The landscape areas with good resilience are mainly the wetland of the Xilin River Basin and the cultivated land.

### 3.5. Ecosystem Services

**Figure 8:**
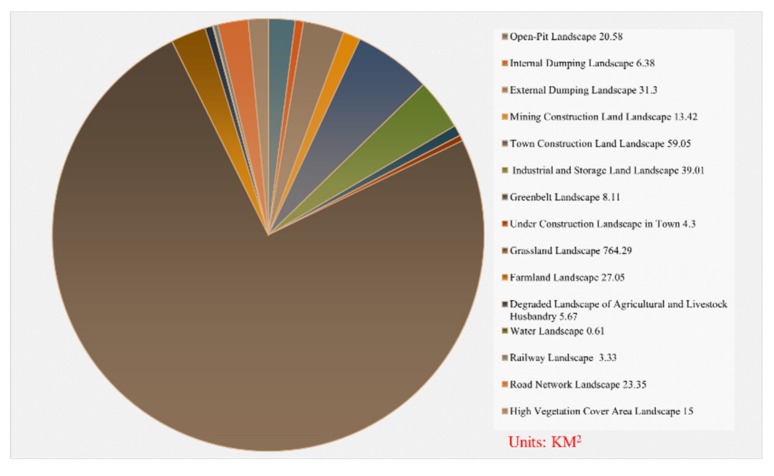
The area of various landscapes.

**Figure 9:**
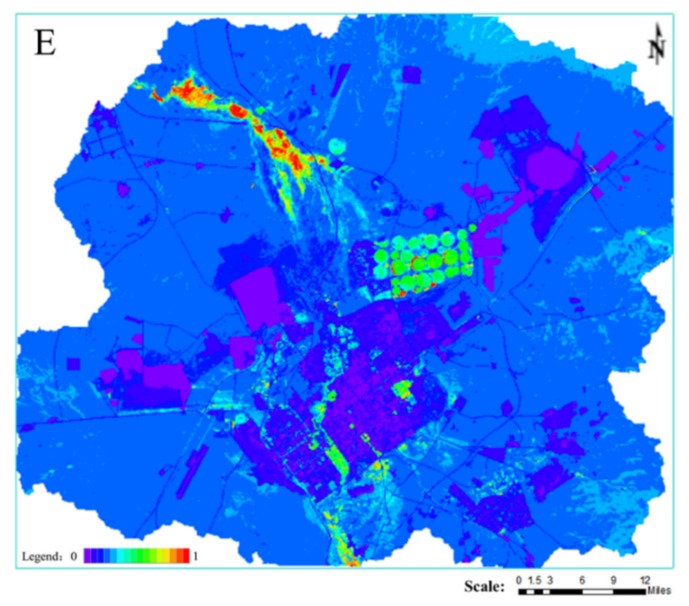
Spatial distribution of ecosystem services.

According to the landscape ecological classification map (Figure 2) of Xilinhot and its statistical analysis results, the area of the various landscapes is shown in Figure 8. These results show that the study area may be characterized as a grassland-matrix landscape. Besides the grassland landscape, the urban landscape and the mining landscape occupy most of the area. Although the study area belongs to the Xilin River Basin, the water landscape is very small and the drought conditions are evident. In Figure 9, the higher the value, the higher of the ecosystem services. As illustrated in Figure 9, the Xilin River Basin, the cultivated land, and the greenland have higher values of ecosystem services, while the open-pit mine, the industrial storage land, and the urban construction land have lower values.

### 3.6. Landscape Ecological Health Assessment Result of the Mining City

**Figure 10:**
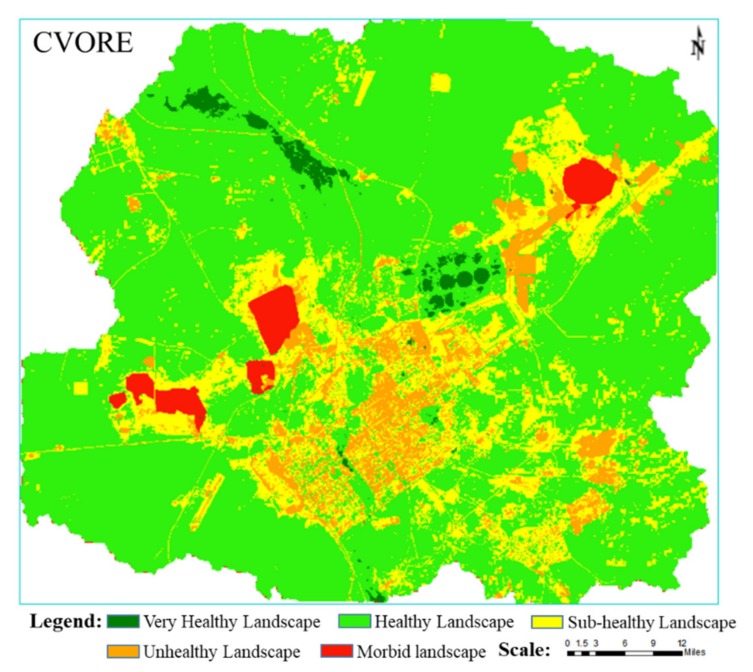
Spatial distribution of landscape ecological health.

The natural breakpoint method was used to classify the LEH of the study area into five levels: very healthy (>0.6); healthy (0.4–0.6); sub-healthy (0.3–0.4); unhealthy (0.15–0.3); and morbid landscape (<0.15). As illustrated in Figure 10, the very healthy landscapes are mainly located in the Xilin River Basin wetland, the cultivated land, and the green land. Healthy landscapes account for the majority of the total area, and most grasslands are healthy. The sub-healthy landscapes are mainly located around areas with higher human disturbance, such as mining areas, towns, industrial buildings, airports, roads, and railways. In addition, the artificial dump in mining areas is in a sub-healthy state. Unhealthy landscapes are mainly located in urban, mining, and industrial storage construction sites. Morbid landscapes are mainly located in the open-pit. Thus, it can be seen that the morbid or unhealthy landscapes are concentrated in the mining production and urban living areas, while the landscape gradually becomes healthier from the center to the outside. Accordingly, the health level gradually transits towards unhealthy, sub-healthy, and healthy states. The health distribution follows a pattern of “less at both ends, more in the middle”. Very healthy, morbid, and unhealthy areas are smaller, while healthy and sub-healthy areas are larger. The area and the proportion of the extension of the different health levels are shown in Table 2.

## 4. Discussion

### 4.1. Effects of Mining and Water Landscape on Landscape Ecological Health of Grassland

**Figure 11:**
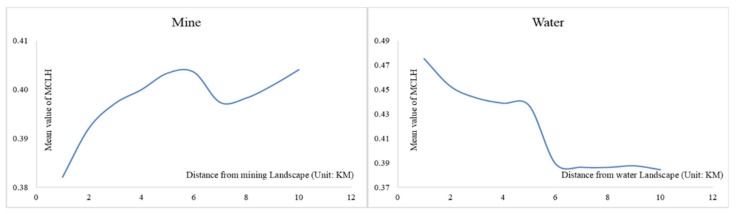
Buffer Analysis of Grassland LEH around Mining and Water Landscape.

It can be seen from Figure 10 that the grasslands surrounding the areas with more human disturbances such as mining areas and cities are basically in a sub-healthy state, while the areas around the Xilin River are in a very healthy state. In order to further study the impact of mining and water on grassland LEH, this study used 1 km as buffer analysis step to analyze the LEH status of grassland around the mining landscape and water landscape respectively. It can be seen from Figure 11 that the farther away from the mining landscape, the higher the average value of grassland LEH; The farther away from the water landscape, the lower the average value of the grassland LEH. Thus, mining has a significant negative impact on grassland LEH, while water has a significant positive impact on grassland LEH. Soil and water conservation is extremely important for grassland ecological protection.

### 4.2. Advantages of the CVORE Comprehensive Index Model

**Figure 12:**
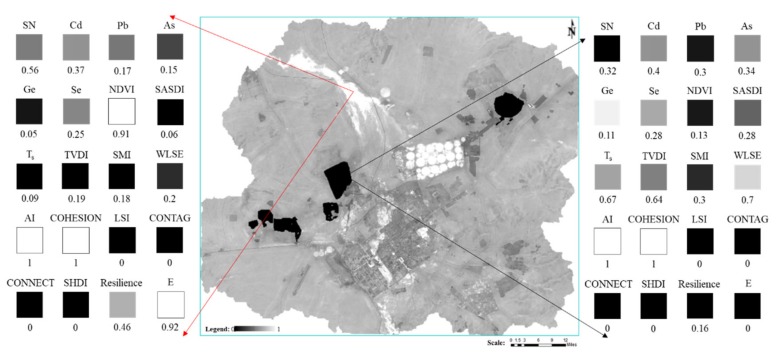
Analysis of landscape ecological health problems at any two points.

In this study, a LEH assessment model was constructed through a comprehensive index method. The model has several advantages. First, the model’s Vigor, Organization, Resilience, and Ecosystem services are all based on real-time data such as remote sensing images, DEM, and meteorology data. Without considering the Condition, a VORE model can be easily constructed to monitor and assess the LEH of mining cities in real time. The difficulty in using this model lays in the fact that a considerable amount of manpower and of materials are needed to collect and test the basic condition index. Therefore, it is necessary to develop satellite hyperspectral remote sensing technology to obtain soil parameters in real-time and in a quantitative way for regional LEH monitoring and environmental protection. Second, as shown in Figure 1, from bottom to top, a CVORE comprehensive index system assessment model can be constructed, which can assess the health status of the study area in time and space and answer to questions such as: which region is not healthy? From top to bottom, it is possible to identify the main factors affecting the health of any region (see Figure 12). In this way, it is possible to formulate ecological restoration strategies. Third, the model considers the characteristics of the semi-arid steppe, such as drought, salinization, desertification, low vegetation coverage, poor ecological resilience after disturbance, high heterogeneity of ecosystem services, and the characteristics of WLSE, SN decline, heavy metal pollution, landscape fragmentation, and connectivity decline in mining city. In this way, the model advances a new way to select the LEH assessment index system and to construct the model for the semi-arid steppe mining city. Fourth, the CVORE model constructed in this study has the advantage of being flexible, open, and inclusive. When assessing the LEH of another mining city, the assessment index system can be flexibly constructed under the framework of the CVORE model according to the characteristics of other research areas.

### 4.3. Research Prospects of Landscape Ecological Health in Mining Cities

Although the concept of LEH has been put forward for decades, there are still many immature theories and technical methods. Several problems are present in the ecological health of mining cities, which need further research and resolution. They are briefly discussed below:

(1) *Selection of indexes*. The indicators used in this study are more focused on remote sensing images, which are easily and dynamically captured in real time. However, the LEH of mining cities should also include other aspects like animal communities, plant communities, microbial communities, hydrology, geology, and atmosphere. Researchers should monitor these ecological factors over a long time period and observe their dynamic changes throughout the life cycle of mining, as well as the relationship between mining and these ecological factors. At the same time, researchers should also investigate the dynamic changes of human health, social stability, public health, economic structure, and of other factors during the whole life cycle of mining, as well as the relationship between mining and these socio-economic factors.
(2) *Research on influence mechanism(s)*. This study is based on data covering only the year 2017. Further research should investigate the impact mechanism of mining exploitation on LEH, the driving forces of LEH evolution in mining cities, the impact of landscape processes on LEH, and the impact of landscape pattern on LEH, through a time series LEH assessment.
(3) *Scale problem*. The LEH assessment of mining cities should focus on three core scales: mining city, mining groups, and mining areas. At the scale of the mining city, it should include at least grassland landscapes, town landscapes, mining landscapes, and other spatial scales with clear differences. Although the results of LEH assessment at different spatial scales are closely related, the non-deductibility of scale determines that LEH at larger scales cannot be linearly reduced to smaller scales, nor is it a simple accumulation of ecological health at smaller scales. Therefore, the LEH assessment of mining city should be performed at multiple scales. At the same time, although the research objects of LEH at different scales have different emphasis, they are not completely separated from each other and are closely related. The comprehensive study of the coordination and the organic integration of LEH at multiple scales, and the ways and methods of transformation among different scales (i.e., multi-scale synthesis and scale conversion of evaluation results), are the key scientific issues of LEH comprehensive research in large mining city [57,58].

### 4.4. Suggestions on Landscape Ecological Restoration for Mining Cities in Semi-Arid Steppe

The goal of mine ecological restoration is to maintain a healthy landscape ecosystem and to establish a harmonious relationship between humans and nature [59]. Therefore, the landscape ecological restoration of steppe mining cities is based on the grassland LEH as the starting point (through the landscape ecological investigation and health assessment); and on the theoretical basis of the mechanism of the impact of coal exploitation on the steppe LEH. The aim is to identify the key parts or strategic components of regional landscape ecological process and function, targeted for landscape ecological restoration. By combining a series of restoration strategies at the three scales of mining city, mining group, and mining area, a landscape ecological restoration system of mining city can be formed, and the ideal restoration effect can be achieved:

(1) *Mining city scale*. Coal exploitation has affected the sustainable and healthy development of steppes. Due to the excavation, occupied, and piled up areas, healthy landscape patches are reduced, the landscape patches are fragmented, the landscape connectivity decreases, the dimension of habitat patches is reduced and gradually islands form. This in turn seriously hinders the proliferation and circulation of species, while at the same time interfering with the landscape’s own regulatory capacity, and resulting in the reduction of local biodiversity and ecosystem services. Through simulating and controlling the process of WLSE and the biogeochemical cycle, the objectives and standards of landscape ecological optimization were established. Moreover, the spatial and quantitative distribution and connectivity of landscape components were optimized by low-impact development and the development of a network of green infrastructure. In this way, it was possible to maintain the regional LEH within the best possible pattern. As shown in Figure 10, ecological corridors can be constructed to increase landscape connectivity in areas with severely fragmented landscape health patches (as in the southeastern side of the study area), thus increasing the selection of corridors for species migration and the possibility of material and energy flows. In addition, the diseased, unhealthy, and sub-healthy landscapes caused by the presence of the mining area, the city, and the road have been linked together. For this reason, it is necessary to set up landscape isolation zones between the city and the mining area. The existing windbreak forest can be used as the core, while shrubs with a long growth period and a strong ability to suppress wind and dust can be selected to transform the existing windbreak belt. On the one hand, the healthy landscape forms a network; on the other hand, it blocks the unhealthy landscape and the dust.
(2) *Mining group scale*. Steppe mining cities involve the large-scale continuous mining of multiple mines for a long period of time. The restoration of landscape ecology must be based on the understanding of the comprehensive impact of mine exploitation on the LEH of steppe mining city, and on the control of the entire situation from three perspectives: space, time, and ecological environment. First, at the spatial level, a large number of permanent excavation sites, mining pits, internal dumping sites, and other industrial and mining landscape units are optimized to the spatial pattern and landform morphology. At the same time, new landscapes such as ecological green belts and temporary wetland units are introduced, improving and controlling WLSE, surface hydrological process, and material cycle and pollutant diffusion in the mining city. Second, in the time dimension, the life cycle of mining should be understood. This means that the simulation, analysis, and design of mine landscape ecological restoration should be combined with mining planning to formulate the whole life cycle of the landscape ecological restoration plan. In this way, it is possible to avoid the discontinuity of the landscape ecological restoration plan at each stage. Third, in order to protect the integrity and continuity of regional landscape ecology, it is necessary to consider not only the soil reconstruction, land renovation, and vegetation construction of mine landscape units such as the dump, but also the integration of the landscape with the surrounding natural landforms. In this way, the connectivity between the dump and the surface water hydrology in surrounding areas will be improved.
(3) *Mining scale*. Open-pit mining damages the original surface, resulting in the deterioration of LEH and in the formation of large-scale artificial padding landscapes. Soil compaction is caused by heavy machinery operations; soil and vegetation functions are seriously impaired and, under extreme weather conditions, rock and WLSE in mining areas is intensified. Traditional reclamation methods often form a flat horse surface; the water system is not mature enough and can be easily eroded by natural concentrated rainfall; finally, erosion is more serious under extreme climate conditions. Moreover, it is difficult to maintain biodiversity in a single geomorphic environment, which limits the rapid restoration of the ecological environment to resist desertification and erosion. Therefore, it is necessary to maintain the stability and ecological sustainability of the geological environment of the internal/external dump and of the mining pit in the mining area, to control their impact on the surrounding landscape matrix or patch habitats, and to enhance their integration with the surrounding grassland natural landform and hydrology through the life cycle near-natural design. The restored landscape should be coordinated with its surroundings; the slope surface should be kept stable for a long time; the soil and water conservation should be maximized; and the original ecological status should be restored for soil microorganisms, vegetation, and animals. Without additional soil and water conservation measures, no runoff will occur on the slopes. The winding drainage channels and the continuous catchment pits allow all the rainwater to infiltrate and accumulate in the watershed. In this way, the restored ecosystem can maintain itself and gradually enhance its function.

## 5. Conclusions

At present, the study of LEH is still in the initial stage of development, even though, being a new method of environmental management, it is receiving increasing attention by researchers. A healthy landscape ecosystem is increasingly recognized as the ultimate goal of environmental management. Because the LEH of mining cities is a spatiotemporal distribution problem, the comprehensive application of Remote Sensing (RS), Geographic Information System (GIS), and Global Positioning System (GPS) 3S technology to quickly obtain and accurately analyze the basic data in order to dynamically monitor the macroscopic LEH status, has become the objective requirement of LEH quantitative assessment. This study used the Xilinhot as an example to construct a CVORE model, aiming at a scientific and comprehensive assessment the LEH of mining city. The CVORE model proposed in this study takes into account the characteristics of semi-arid steppes, such as drought, salinization, desertification, low vegetation coverage, poor ecological resilience after disturbance, high heterogeneity of the ecosystem services, and the characteristics of WLSE, SN decline, heavy metal pollution, landscape fragmentation, and connectivity decline in mining city. Moreover, the CVORE model has the advantage of being flexible and open. When assessing the LEH of another mining city, the assessment index system can be flexibly constructed under the framework of the CVORE model, according to the characteristics of each research area. The research results show that the area of very healthy, healthy, sub-healthy, unhealthy and morbid landscape are 13.23, 736.35, 184.5, 66.76 and 20.63 km^2^, respectively. The very healthy landscapes are mainly located in the Xilin River Basin wetland, cultivated land, and green land. Healthy landscapes account for the majority of the total area, and most grasslands are healthy. The sub-healthy landscapes are mainly located around areas with higher human disturbance, such as mining areas, towns, industrial buildings, airports, roads, and railways. The morbid or unhealthy landscapes are concentrated in the mining production and urban living areas. This study will provide a reference for LEH assessment research, landscape ecological restoration, environmental protection and urban planning of semi-arid steppe mining cities.

## Figures and Tables

**Table 1:** The landscape ecological significance of each landscape pattern indices.

Name	Abbreviation	Landscape Ecological Significance
Aggregation index	AI	The AI is used to indicate the probability of appearance of different patches on the landscape map. The AI value increases with the increase of the aggregation degree.
Patch cohesion index	COHESION	As connectivity decreases, COHESION decreases.
Connection index	CONNECT	As the connectivity between patches increases, the value of CONNECT increases.
Contagion index	CONTAG	The CONTAG index is used to measure the ratio between the observed spread and the maximum possible spread under a given patch type number. When all patch types are maximally fragmented and intermittently distributed, the index value approaches 0. When the patch type is maximally clustered together, the index reaches 100.
Landscape shape index	LSI	With the increase of LSI, the patch becomes increasingly dispersed and the shape of the patch becomes more irregular.
Shannon’s diversity index	SHDI	In the landscape system, the more abundant the land use, the higher the degree of fragmentation, the more uncertain the information content, and the higher the SHDI value

**Table 2:** Area and proportion of each level of landscape ecological health.

Type	Threshold	Area (km^2^)	Proportion (%)
Very Healthy Landscape	>0.6	13.23	1.30
Healthy Landscape	0.4–0.6	736.35	72.08
Sub-healthy Landscape	0.3–0.4	184.50	18.06
Unhealthy Landscape	0.15–0.3	66.76	6.54
Morbid Landscape	<0.15	20.63	2.02
Total	-	1021.47	100.00
